# Loose Polyester Nanofiltration Membrane Designed with Hydroxyl-Ammonium for Efficient Dye/Salt Separation

**DOI:** 10.3390/membranes15020059

**Published:** 2025-02-10

**Authors:** Nan Ma, Guiliang Li, Yang Liu, Shenghua Zhou, Fu Liu

**Affiliations:** 1 Zhejiang International Joint Laboratory of Advanced Membrane Materials & Processes, Ningbo Institute of Materials Technology & Engineering, Chinese Academy of Sciences, No. 1219 Zhongguan West Road, Ningbo 315201, China; 2 Department of Civil Engineering, The University of Hong Kong, Pokfulam, Hong Kong, China; 3 Ningbo College of Materials Technology & Engineering, University of Chinese Academy of Sciences, 19 A Yuquan Road, Shijingshan District, Beijing 100049, China

**Keywords:** polyester nanofiltration membrane, monomer design, inorganic salts, dye separation, loose network

## Abstract

Efficient dye/salt separation poses a great challenge to nanofiltration (NF) membrane technology in the desalting sector of the dye synthesis industry. In this study, we fabricated a novel loose polyester NF membrane via an interfacial polymerization method using “hydroxyl-ammonium” biquaternary diethanolamine (MDET) and trimesoyl chloride. The molecular design of MDET provides a loose crosslinking network, showing high rejection of dyes and the passage of monovalent salt/divalent salt ions in the dye solution, exhibiting exceptional filtration efficiency with high selectivity. Furthermore, the membrane exhibits excellent operational stability for over 100 h, demonstrating superior antifouling properties and high resistance to chlorine. This study provides new insights into the role of dyes and mono- and divalent ions in desalination processes related to the dye synthesis industry.

## 1. Introduction

Synthetic dyes, as indispensable raw materials, have permeated a myriad of industries, playing an important role in various industries such as rubber production, textile manufacturing, leather tanning, cosmetics formulation, and pharmaceutical development [1,2,3]. Globally, the annual consumption of synthetic dyes surpasses an astounding 700,000 tons, with the textile sector claiming over half of this substantial quantity [4,5]. Desalination is a critical process in dye production, where inorganic salts such as Na_2_SO_4_, MgSO_4_, NaCl, MgCl_2_, etc., serve as essential additives. However, inorganic salts at concentrations as high as 30% pose a significant challenge to achieving high-purity dyes [6,7]. Traditional purification technologies, such as evaporation and solvent extraction, demand substantial energy consumption and solvent usage, leading to high costs and inefficiencies [8,9]. Membrane technology represents a promising candidate due to its lower energy consumption for separation processes and the absence of chemical or biological changes to the products [10,11].

Among them, nanofiltration (NF) membranes, as a rapidly developing technology, feature nano/sub-nano pore sizes and surface charges that enable the effective separation of molecules and ions through solution diffusion, size exclusion, or electrostatic repulsion [12,13]. However, the dense selection layer impedes the fast transport of ions. An ideal high-performance NF for efficient concentration should ensure high retention of dyes while facilitating the permeation of inorganic salts [14]. Increasing the free volume within the selection layer for dye desalting was found to be an effective strategy in [15]. By developing a loose nanofiltration (LNF) membrane with an enhanced permeable active layer and larger pore sizes, the membrane exhibited high permeation rates for salts and a molecular weight cut-off (MWCO) range of 500–2000 Da, thereby achieving superior monovalent and divalent salt ion permeability [16,17,18]. The fabrication process of LNF has employed various preparation techniques, including surface modification, layer-by-layer self-assembly, co-deposition, and nano-filler doping [19,20]. However, these methods frequently entail intricate procedures and yield suboptimal outcomes. Specifically, they necessitate the application of multiple modification techniques and exhibit low permeability to multivalent salts, particularly common divalent salts, thereby hindering the effective separation of salt and dye molecules.

Rational monomer design is anticipated to address the above-mentioned issues. Yet, it is also crucial to consider the potential membrane fouling caused by dyes present in the separation, as well as the stability of the membrane structure during subsequent chemical cleaning processes [21]. Compared to the traditional polyamide NF membrane, the emerging polyester membrane exhibits enhanced resistance to pollution (derived from high hydrophilicity) and chlorine (due to the absence of active hydrogen atoms), which is attributed to the presence of polyester bonds [22,23]. In addition, compared to amino monomers, hydroxyl monomers exhibit relatively lower reactivity, which facilitates the formation of polyester selective layers with lower cross-links and larger pore sizes during interfacial polymerization (IP) with acyl chloride monomers. For instance, Jin et al. [24] prepared a polyester NF membrane with excellent water permeability and high transmission of salts. In addition, Xue et al. [25] developed a polyester NF membrane through an IP reaction between cyclodextrin containing -OH groups and trimesoyl chloride (TMC), which exhibited superior chlorine resistance and made it suitable for textile wastewater treatment.

**Figure 1:**
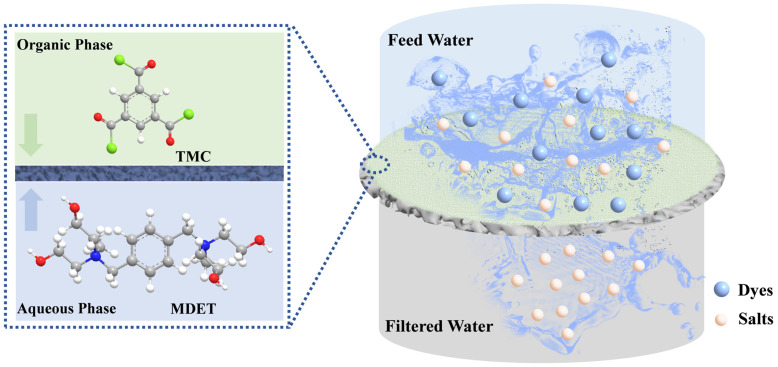
Schematic illustration of loose polyester nanofiltration membrane for efficient dye desalination.

In this study, we synthesized a novel biquaternary diethanolamine (MDET) featuring a biquaternary ammonium group within the benzene ring and a terminal 4-OH group on the flexible alkyl chain. This compound was used as the primary building block for fabricating ultra-thin and smooth LNF membranes. The synthesized monomer combines rigid and flexible segments, resulting in a selective layer with a high free volume. The large pore size and low crosslinking facilitate efficient permeation of inorganic salts, including monovalent/divalent salt ions, while maintaining the interception of dyes. Additionally, the polyester structure ensures excellent chlorine resistance (Figure 1). This work provides valuable insights into the design of highly selective and stable nanofiltration membranes for dye desalination.

## 2. Materials and Methods

### 2.1. Materials

Polysulfone (PSF) supporting membranes (MWCO: 30 kDa) were acquired from Hunan Keensen Technology Co, Ltd. (Changsha, China). N-Methyldiethanolamine (MDEA, 98%), acetonitrile (99.8%), trimesoyl chloride (TMC, 98%), sodium hydroxide (97%), all inorganic salts (MgSO_4_, MgCl_2_, Na_2_SO_4_, NaCl), and bovine serum protein (BSA) were bought from Aladdin Co., Ltd. (Shanghai, China). α, α′-Dibromo-p-xylene (97%), N-hexane, sodium dodecyl sulfate (SDS), and anionic dyes such as Sunset Yellow FCF (SY, 452 Da), Congo Red (CR, 696 Da), Brilliant Blue (BB, 792 Da), and Evans Blue (EB, 960 Da) were purchased from Macklin Biochemical Co., Ltd. (Shanghai, China). All the aforementioned reagents were used without further purification.

### 2.2. Synthesis of the MDET Monomer

**Figure 2:**
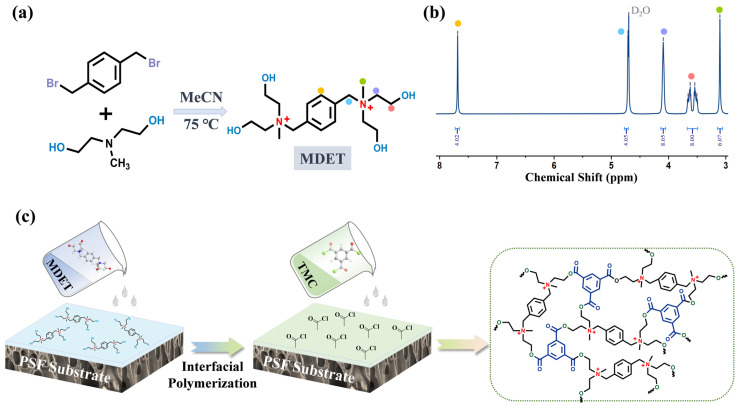
(**a**) The synthesis and (**b**) ^1^H NMR spectra of MDET, and (**c**) the fabrication scheme of the MDET-TMC TFC membranes.

α, α′-Dibromo-p-xylene (10 mmol, 2.6 g) was dissolved in 60 mL of acetonitrile in a three-necked flask and stirred in an oil bath at 75 °C. Subsequently, the initial monomer MDEA (60 mmol, 6.7 g) was added to the reaction mixture under continuous stirring, the mixture was maintained at 75 °C for 12 h. The resulting white precipitates were collected after the reaction was over, then washed several times with acetonitrile to remove unreacted monomers, and dried in a vacuum oven at 60 °C for 12 h to obtain the final product MDET. The synthesis pathway of MDET is illustrated in Figure 2a,b.

### 2.3. Fabrication of MDET-TMC Thin-Film Composite (TFC) Membranes

The polyester LNF membrane was prepared via IP between MDET and TMC on the PSF UF membranes. The fabrication procedure and molecular structure of the reactants are illustrated in Figure 2. Firstly, PSF substrates were immersed in distilled (DI) water to eliminate impurities on the surface. After rinsing with DI water and drying, the MDET aqueous solution with different concentrations (10 mL, containing 0.05 wt.% SDS, pH = 13.5) was brought into contact with the clean polysulfone (PSF) substrate surface for 3 min. After this, we dried the excess solution with filter paper, and it underwent natural air-drying at room temperature (25 °C) for 5 min. Secondly, the organic phase TMC/n-hexane solution (5 mL, 0.3 wt.%) was gently poured onto the MDET-saturated PSF substrate to initiate the IP reaction at 25 °C for 1 min. Subsequently, the remaining TMC solution was removed from the membrane surface using a large amount of hexane. The resulting MDET-TMC TFC membrane (TFCM) (Figure 2c) was further heat-treated in an oven at 60 °C for 10 min. Additional membranes prepared with MDET concentrations of 1 wt.%, 1.5 wt.%, 2 wt.%, and 2.5 wt.% were designated as M_1_, M_2_, M_3_, and M_4_, respectively. The IP temperature was room temperature (25 °C).

### 2.4. Characterizations of Membranes

The morphology of the membrane cross-section and surface was examined using transmission electron microscopy (TEM, JEOL2100, Tokyo, Japan) and field emission scanning electron microscopy (FESEM, S-4800, Hitachi, Tokyo, Japan). The structure of the MDET monomer was analyzed via nuclear magnetic resonance spectroscopy (^1^H NMR, AVANCE III 400 MHz, Bruker, Billerica, MA, USA). Atomic force microscopy (Bruker, Billerica, USA) was employed to determine the root-mean-square (RMS) roughness of the membranes. Fourier transform infrared spectroscopy (FTIR, NICOLET 6700, Thermo Fisher Scientific, Waltham, MA, USA) and X-ray photoelectron spectroscopy (XPS, Axis Ultra DLD, Kratos, London, UK) were employed to analyze the functional groups and chemical composition of the polyester membranes. The Zeta potential of the membrane surface was assessed using a ζ-potential analyzer system (SurPASS, Anton Paar, Graz, Austria) in a 1.0 mmol/L KCl solution at ambient temperature for solid samples. The pH testing range of the KCl solution ranged from 3 to 10 and was adjusted by HCl and KOH solutions.

### 2.5. Separation Performance Tests

Nanofiltration performance tests were conducted in cross-flow filtration devices at room temperature. The practical testing area of the device was 3.14 cm^2^. The outlet flow rate measured 50 cm/s. The operation mode was permeate circulation to the feed tank. All membranes were pre-pressurized with 1 L feed solution (i.e., distilled (DI) water, 1000 mg/L salt solutions, or 50 mg/L dye solutions) at 5 bar for 1 h before the test, and all the filtration tests were carried out under the same pressure. The concentrations of salts (including Na_2_SO_4_, MgSO_4_, MgCl_2_, and NaCl) and dyes (including SY, CR, BB, and EB, which had maximum adsorption wavelengths at 481 nm, 497 nm, 629 nm, and 610 nm, respectively) in the feed solution were 1000 mg/L and 50 mg/L, respectively.

The water flux (J, L m^−2^ h^−1^) was calculated according to Equation (1):

(1) $$J=\frac{V}{A\times \Delta t}$$

where V (m^3^) is the volume of permeate water, A (m^2^) is the effective membrane area, and ∆t (h) is the filtration time.

The rejection (*R*) was calculated by using Equation (2):

(2) $$R=(1-\frac{C_{p}}{C_{f}})\times 100\%$$

where *R* represents the salt and dye rejection (in %), while *C_p_* and *C_f_* denote the solute concentrations in the permeate and feed solutions, respectively. In the experiments, salt concentration was determined using conductivity measurements, while dye concentration was measured with a UV-Vis absorption spectrometer.

### 2.6. Dye/NaCl Separation and Stability Performance Evaluation

Dye/NaCl separation using MDET-TMC TFC M_3_ was carried out in the same cross-flow filtration devices at 5 bar. Mixed solutions containing dye (50 mg/L) and NaCl (1000 mg/L) were used as feed solutions (pH = 7) to evaluate the dye/NaCl separation performance. We measured the concentrations of dyes and salts in both the feed solution and the permeate simultaneously every hour. The separation factor (*S*) was determined by Equation (3):

(3) $$\;S=(1-R_{NaCl})/(1-R_{dye})$$

where $R_{NaCl}$ and $R_{dye}$ are the rejection of NaCl and dye, respectively.

The stability of the performance of the TFC M_3_ was studied through a filtration test. A solution containing 1000 mg/L Na_2_SO_4_ was filtered for 100 h by the cross-flow filtration devices at 5 bar. Then, water flux and solution rejection were measured as mentioned before.

### 2.7. Antifouling Performance Measurements

The membrane antifouling performance was tested using BSA (100 mg/L) solution and EB (50 mg/L) solution as representative pollutants. Initially, the membrane was conditioned by filtering distilled (DI) water for 60 min at 5 bar to achieve a steady state, and pure water flux was recorded every 30 min as $J_{0}$. Afterward, the ultrapure water was replaced with the BSA solution or EB solution to perform the anti-fouling experiment. In each cycle for continuous filtration with BSA or EB pollutant, the flux for BSA or EB solution was recorded as $J_{w}$. Finally, the polluted membrane was flushed with DI water for 60 min and the recovered water flux after cleaning the membrane was denoted as $J_{r}$. The correlation indices for antifouling properties were calculated using Equation (4) and Equation (5), respectively:

(4) $$FRR=\frac{J_{r}}{J_{0}}\times 100\%$$

(5) $$FDR=\frac{J_{0}-J_{w}}{J_{0}}\times 100\%$$

where *FRR* (%) and *FDR* (%) represent the flux recovery ratio and fouling decline ratio, respectively.

### 2.8. Chlorine Resistance Performance

The resulting MDET-TMC TFC M_3_ was dipped into sodium hypochlorite solution (500 mg/L pH:7) for 50 h and 100 h. Afterwards, the membranes were rinsed and stored in DI water. The water flux and the rejection of dye and salt were examined using the same cross-flow filtration devices at 5 bar. The performance of the membrane was measured every 50 h.

## 3. Results and Discussion

The successful synthesis of the MDET monomer was analyzed using the the ^1^H NMR spectrum in Figure 2b. Five peaks were observed at δ 7.68 ppm (4H, Ar-**H**), δ 4.71 ppm (4H, ArC**H**_2_), δ 4.09 ppm (8H, N^+^C**H**_2_), δ 3.61 ppm (8H, C**H**_2_OH), and δ 3.10 ppm (6H, N^+^C**H**_3_) (Figure 2b). The ratio of the areas of the five peaks in MDET was determined to be 2:2:4:4:3, which is consistent with the symmetric structure of MDET.

**Figure 3:**
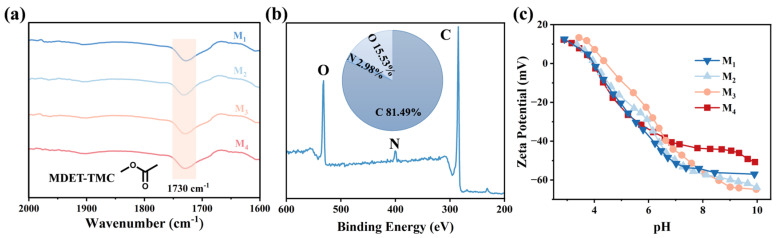
(**a**) FTIR spectra and (**b**) XPS spectra of MDET-TFC TFCMs. (**c**) Surface zeta potential of MDET-TMC TFC membranes.

The chemical composition of the membrane surface was characterized using XPS and FTIR. Figure 3a shows the FTIR spectra, where all membranes exhibit a prominent peak at 1730 cm^−1^, attributed to the ester bond formed from the “hydroxyl-acryl” reaction. Further, the elemental compositions of the MDET-TFC membranes were determined by the XPS measurements. As shown in Figure 3b, the shoulder peak at 400.8 eV is more clearly seen, which was assigned to quaternary ammonium (-N^+^). Based on the XPS characterization, the C/N molar ratio of the MDET-TFC TFCMs is determined to be 27.3. The calculated MDET/TMC involved in the reaction is 0.23. The aforementioned results substantiate the stoichiometric condensation of MDET-TFC TFCMs at the interface.

Figure 3c shows the surface zeta potentials of the TFC membranes synthesized from different aqueous monomer concentrations. The MDET-TMC membranes exhibited a significantly negative charge density at neutral pH. Furthermore, the negative charge of the resulting MDET-TMC membranes weakened in an orderly manner with increasing MDET incorporation, mainly attributed to the reduction in residual chloride groups. Concurrently, the incorporation of the positively charged quaternary ammonium groups neutralized a portion of the negative charges and weakened the electronegativity on the surface of the MDET-TFC membrane.

**Figure 4:**
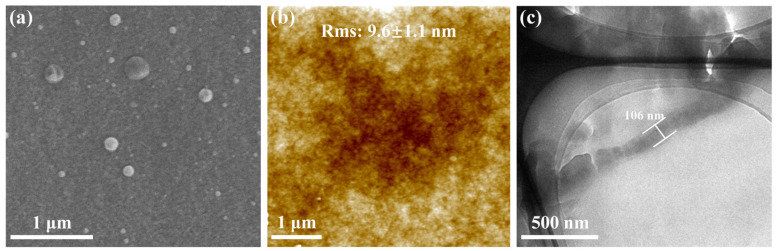
(**a**) The SEM surface morphology, (**b**) AFM image, and (**c**) TEM morphology of the MDET-TFC membrane (M_3_).

TFC membranes were fabricated via an IP reaction on polysulfone substrates. As shown in Figure 4a, the MDET-TMC TFC membrane exhibits a relatively smooth surface, which was further confirmed by AFM (Figure 4b) analysis, revealing a surface roughness of 9.6 ± 1.1 nm. Lower roughness is advantageous for minimizing the adhesion of pollutants to the membrane surface. High-resolution TEM analysis of the ultrathin slice-treated membrane indicated that the MDET-TMC polyester selective layer has a uniform thickness of approximately 106 nm.

**Figure 5:**
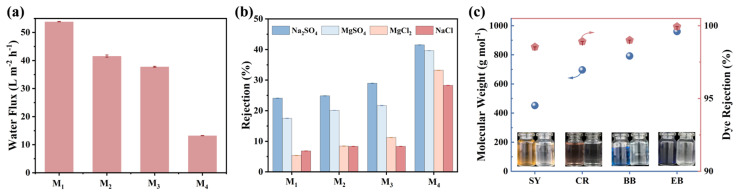
(**a**) Water flux, (**b**) single salt rejection, and (**c**) single dye rejection of the M_3_ membrane. The operating pressure is 5 bar, and the concentrations of the single salt solution and the single dye solution are 1000 mg/L and 50 mg/L, respectively.

Figure 5 demonstrates the separation efficiency of the MDET-TMC TFC membranes. We systematically investigated the concentration of MDET in the system, which played a pivotal role in selective layer formation to determine the optimum conditions for synthesizing high-selective-performance MDET-TMC TFC membranes for dye removal. Initially, under neutral pH conditions, we examined the water flux and selectivity (using targeted salt ions and dye molecules including Na_2_SO_4_, MgSO_4_, MgCl_2_, NaCl, SY, CR, BB, and EB) as functions of MDET concentration. As the MDET concentration increased from 1 to 2.5 wt.% (Figure 5a), the thicker the polyester layer, the greater the mass transfer resistance of water; thus, the water flux exhibited a continuous decrease from 53.8 L m^−2^ h^−1^ to 13.2 L m^−2^ h^−1^, whereas the salts rejection slightly increased. Among them, the M_3_ membrane exhibited low rejection for all the inorganic salts in the order of Na_2_SO_4_ (28.9%) > MgSO_4_ (21.7%) > MgCl_2_ (11.2%) > NaCl (8.3%) (Figure 5b). The prepared membranes demonstrated low retention of both monovalent and divalent salts. This phenomenon was mainly attributed to the fact that the MDET polyester cross-linked networks possessed a relatively loose structure, and thus have great potential to effectively remove these salts from the unpurified product. At low concentrations of MDET, such as 1 wt.%, the availability of hydroxyl groups during IP is limited, leading to the formation of a relatively loose cross-linked selective layer. As the MDET concentration increases to 2.5 wt.%, more hydroxyl groups participate in the IP process, resulting in greater consumption of acyl chloride groups and the formation of a denser selective layer. However, compared to other NF membranes, this selective layer remains relatively loose [26,27]. Furthermore, the increased MDET concentration not only thickened the selective layer but also narrowed the pore size distribution and enhanced crosslinking. These microstructural changes significantly elevated the mass transfer resistance, thereby contributing to the observed decrease in water flux and an increase in the rejection performance of the MDET-TMC TFC membranes.

Generally speaking, during the dye synthesis process, inorganic salts are either added or generated as part of the reaction. Therefore, a series of anionic dye molecules as modules with different molecular weights were selected to test the universality of the membrane’s rejection effect. In this study, four dye molecules, SY, CR, BB, and EB, were used to investigate the potential application of MDET-TMC TFC membranes in the process of dye removal. As shown in Figure 5c, unlike salt ions of small size, MDET-TMC TFC membranes exhibited a stable ability to retain negatively charged dyes at large membrane pore sizes, with an impressive rejection rate of over 98.5%. The separated solution exhibited an almost colorless appearance.

**Figure 6:**
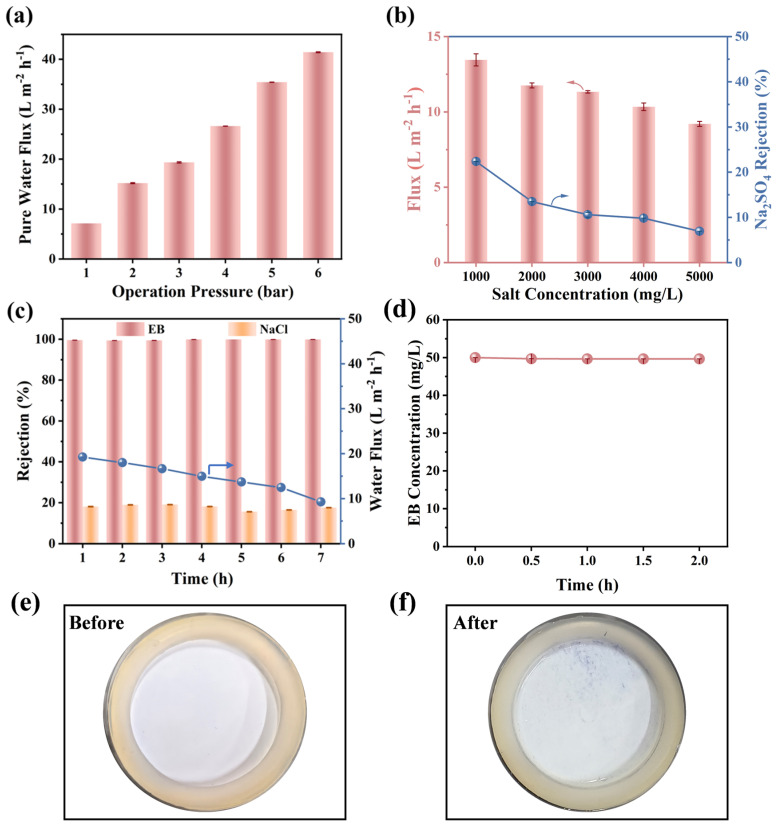
(**a**) Operation pressure in pure water flux and (**b**) Na_2_SO_4_ rejection of M_3_ membranes (1000 mg/L Na_2_SO_4_ as feed solution). (**c**) Separation of EB/NaCl mixed solution (EB: 50 mg/L, NaCl: 1000 mg/L) using M_3_ membranes. (**d**) EB concentration in feed during two-hour operation (EB: 50 mg/L). Picture shows membrane top surface (**e**) before and (**f**) after 7 h of EB filtration.

To investigate the application of as-prepared loose MDET-TMC membranes, the performance of the membrane was further evaluated using a mixed solution containing EB (50 mg/L) and NaCl (1000 mg/L). It is well established that the Donnan effect and steric hindrance synergistically influence the selectivity of NF membranes. Figure 6a illustrates that the water flux of the M_3_ increased gradually as the operating pressure rose from 1 to 6 bar. The efficiency of dye purification in untreated production is influenced by varying salinity requirements, prompting an examination of the impact of salt concentration on membrane separation performance. As the feed Na_2_SO_4_ concentration increased from 1000 to 5000 mg/L (as shown in Figure 6b), solutions with low salt concentrations exhibited low rejection (<30%) for Na_2_SO_4_, and increasing the salt concentration of the feed solution resulted in a further decrease in rejection rates. The water flux of the TFC membrane predictably decreased due to the elevated osmotic pressure [28,29]. These findings suggest that TFC M_3_ exhibits excellent performance in permitting the permeance of both divalent and monovalent salts across a wide range of salt concentrations.

In order to effectively separate dyes with salts from a dye/salt mixture during the treatment process, the membrane should have a high rejection rate for dye molecules, as well as high fluxes for salt. As shown in Figure 6c, TFC M_3_ exhibited a gradual decrease in water flux while achieving nearly complete rejection (7 h, *R_EB_* > 99.9%) of the larger dye molecules such as EB, alongside modest rejection (<20%) of smaller NaCl. This separation capability holds significant potential for producing dyes with enhanced purity. Furthermore, the high desalination efficiency for EB/NaCl mixtures was consistently maintained over 7 h of continuous operation, with an EB/NaCl separation factor of 825, highlighting the excellent and stable separation performance of the loosely crosslinked MDET-TMC membrane.

Regarding the investigation of the dye adsorption performance of MDET-TMC TFC M3, As shown in Figure 6d, through filtration, the EB concentration in the feed solution remained almost constant with the concentration at the start of the filtration. At the same time, by comparing the photos of the membrane surface before and after the 7 h EB filtration experiment (Figure 6e,f), it can be seen that the dye was not easily adsorbed on the membrane surface. This indicates that MDET-TMC TFC M3 has excellent resistance to dye adsorption.

**Figure 7:**
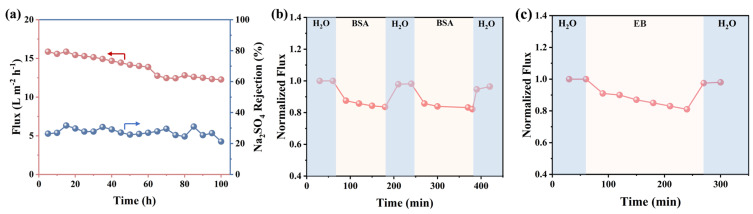
(**a**) Variation in flux and rejection of MDET-TMC TFC M_3_ membrane during 100 h continuous operation at 5 bar pressure with 1000 mg/L Na_2_SO_4_ as feed solution. Antifouling property of TFC M_3_ membrane during filtration of (**b**) 100 mg/L BSA solution and (**c**) 50 mg/L EB solution.

To ensure practical applicability, membranes must exhibit favorable long-term stability and anti-fouling capabilities. The long-term stability of the MDET-TMC TFC membrane was evaluated using a cross-flow filtration system. Specifically, a salt solution containing 1000 mg/L Na_2_SO_4_ was processed at a pressure of 5 bar for 100 h, as illustrated in Figure 7a. During this period, water flux decreased slightly from 15.8 L m^−2^ h^−1^ to 12.2 L m^−2^ h^−1^ while maintaining low retention for Na_2_SO_4_ (<30%). To assess the resistance of the optimized MDET-TMC TFC membrane to organic fouling, dynamic filtration tests were conducted over multiple cycles using a 100 mg/L BSA solution and a 50 mg/L EB solution. As shown in Figure 7b, the normalized flux of the membrane significantly dropped when switching from pure water to the BSA solution. However, hydraulic cleaning effectively restored the fluxes, achieving an *FRR* of 96.4% and an *FDR* of 17.5% in the second cycle. As shown in Figure 7c of the antifouling performance test of the dye EB, the water flux for the MDET-TMC TFC membrane remained constant initially. After replacing the deionized water with the EB solution, there was a significant decrease in flux. The *FRR* for the membrane in the EB solution after deionized water washes was 98.0%, and the *FDR* for the membrane in EB solution for 180 min was 19.0%. This underscores the membranes’ superior anti-fouling properties. Collectively, these findings highlight the potential of these NF membranes for various separation applications.

**Figure 8:**
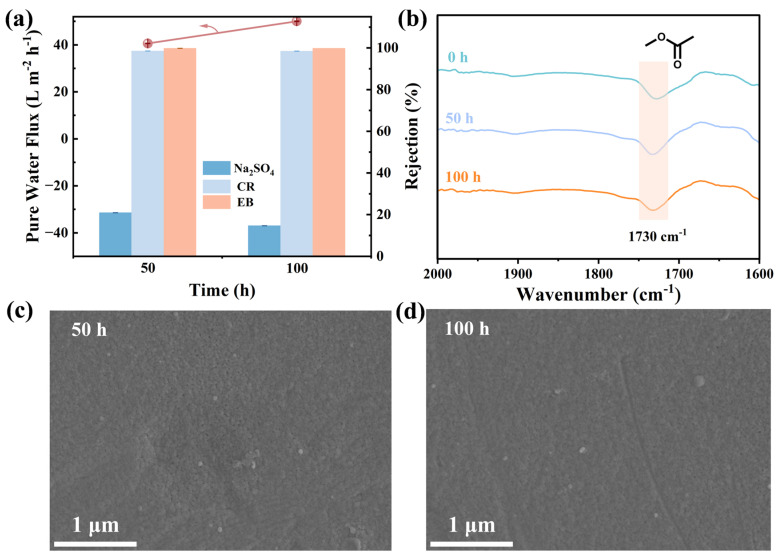
Chlorine resistance of M_3_ exposed to NaClO (500 mg/L, pH = 7) aqueous solution. (**a**) Separation performance including water flux and salt and dye rejection. (**b**) FT-IR spectra. SEM images of the top surface after (**c**) 50 h and (**d**) 100 h (the concentrations of the single salt solution and the single dye solution are 1000 mg/L and 50 mg/L, respectively).

Chemical cleaning is essential for the removal of tenacious membrane fouling, particularly after prolonged operation periods. Subsequently, chlorine stability tests were performed on the MDET-TMC membranes. The membranes maintained steady desalination performance (Figure 8a) over 50 and 100 h at pH ~7.0. As shown in Figure 8b, the FT-IR peaks for ester bonds (1730 cm^−1^) in the MDET-TMC TFC membranes were stable after 100 h of immersion in NaClO aqueous solution. In addition, the MDET-TMC membranes exhibited exceptional oxidative resistance (Figure 8c,d), because their surface morphologies were similar to that of the pristine membrane (Figure 4a). These results show that MDET-TMC membranes are stable in the presence of reactive chlorine.

## 4. Conclusions

In this study, a polyester nanofiltration membrane was fabricated through an IP process, with a newly designed monomer “hydroxyl-ammonium” MDET as the aqueous monomer. The membrane showed loose structures and selective permeability for mono/divalent ions. Consequently, coupled with an appropriate pore size range, the optimal membrane exhibited exceptional separation efficiency (EB/NaCl separation factor of 825 was stable during 7 h of operation), along with a high water permeance of 38 L m^−2^ h^−1^ and low salt rejection (Na_2_SO_4_ = 28.9%, MgSO_4_ = 21.7%, NaCl = 8.3%, MgCl_2_ = 11.2%). The membrane exhibited excellent stability in a Na_2_SO_4_ solution during 100 h of operation, along with notable antifouling properties (*FRR* = 96.4%) and high tolerance to active chlorine. This study offers novel insights into the roles of dyes and mono-and divalent ions in desalination applications.

## Data Availability

The datasets presented in this article are not readily available. The authors do not have permission to share data.
